# Metformin, Rapamycin, or Nicotinamide Mononucleotide Pretreatment Attenuate Cognitive Impairment After Cerebral Hypoperfusion by Inhibiting Microglial Phagocytosis

**DOI:** 10.3389/fneur.2022.903565

**Published:** 2022-06-13

**Authors:** Mengdi Yu, Xiaoying Zheng, Fangyu Cheng, Bei Shao, Qichuan Zhuge, Kunlin Jin

**Affiliations:** ^1^Zhejiang Provincial Key Laboratory of Aging and Neurological Disorder Research, The First Affiliated Hospital of Wenzhou Medical University, Zhejiang, China; ^2^Department of Neurology, The First Affiliated Hospital of Wenzhou Medical University, Zhejiang, China; ^3^Department of Neurosurgery, The First Affiliated Hospital of Wenzhou Medical University, Zhejiang, China; ^4^Department of Pharmacology and Neuroscience, University of North Texas Health Science Center, Fort Worth, TX, United States

**Keywords:** vascular cognitive impairment, metformin, rapamycin, nicotinamide mono-nucleotide, microglia

## Abstract

Vascular cognitive impairment (VCI) is the second leading form of dementia after Alzheimer's disease (AD) plaguing the elder population. Despite the enormous prevalence of VCI, the biological basis of this disease has been much less well-studied than that of AD, with no specific therapy currently existing to prevent or treat VCI. As VCI mainly occurs in the elderly, the role of anti-aging drugs including metformin, rapamycin, and nicotinamide mono nucleotide (NMN), and the underlying mechanism remain uncertain. Here, we examined the role of metformin, rapamycin, and NMN in cognitive function, white matter integrity, microglial response, and phagocytosis in a rat model of VCI by bilateral common carotid artery occlusion (BCCAO). BCCAO-induced chronic cerebral hypoperfusion could cause spatial working memory deficits and white matter lesions (WMLs), along with increasing microglial activation and phagocytosis compared to sham-operated rats. We found the cognitive impairment was significantly improved in BCCAO rats pretreated with these three drugs for 14 days before BCCAO compared with the vehicle group by the analysis of the Morris water maze and new object recognition tests. Pretreatment of metformin, rapamycin, or NMN also increased myelin basic protein (MBP, a marker for myelin) expression and reduced SMI32 (a marker for demyelinated axons) intensity and SMI32/MBP ratio compared with the vehicle group, suggesting that these drugs could ameliorate BCCAO-induced WMLs. The findings were confirmed by Luxol fast blue (LFB) stain, which is designed for staining myelin/myelinated axons. We further found that pretreatment of metformin, rapamycin, or NMN reduced microglial activation and the number of M1 microglia, but increased the number of M2 microglia compared to the vehicle group. Importantly, the number of MBP^+^/Iba1^+^/CD68^+^ microglia was significantly reduced in the BCCAO rats pretreated with these three drugs compared with the vehicle group, suggesting that these drugs suppress microglial phagocytosis. No significant difference was found between the groups pretreated with metformin, rapamycin, or NMN. Our data suggest that metformin, rapamycin, or NMN could protect or attenuate cognitive impairment and WMLs by modifying microglial polarization and inhibiting phagocytosis. The findings may open a new avenue for VCI treatment.

## Introduction

Vascular cognitive impairment (VCI) is a progressive and heterogeneous neurodegenerative disease with mild cognitive impairment to dementia caused by cerebrovascular disease. Its incidence is just lower than Alzheimer's disease (AD) and might surpass the latter in 2050 accounting for 60% of dementia cases (1, 2). Up to now, dementia has become the 5th leading cause of death all over the world, since the number of people with dementia was rising from 20.2 million in 1990 to 43.8 million in 2016 (3). Besides, it has been found that people with dementia would have more problems with their health than their counterparts (4). On the other hand, mild cognitive impairment also needs more attention, while its prevalence was higher than vascular dementia (VD) in old people, and its mortality was similar to AD (5). Importantly, there is no effective therapy to prevent or treat VCI so far, and an urgent need to develop new drugs and tools for the treatment of VCI.

Metformin, an FDA-approved mammalian target of rapamycin (mTOR) inhibitor, is the first-line hypoglycemic drug for treating type 2 diabetes and metabolic syndrome (6). Emerging studies have demonstrated that metformin also exhibits significant multiple anti-aging, anticancer, and anti-cardiovascular disease benefits beyond its current clinical application. For example, metformin could slow conversion to senescence and extend longevity across species by enhancing immune functions, regulating the circadian rhythm, effectively scavenging free radicals, and modulating other hallmarks of aging (7–9). On the other hand, metformin could also have preventive and therapeutic effects for many neurological disorders such as ischemic stroke, Alzheimer's disease (AD), Parkinson's disease (PD), and Huntington's disease (HD) (7), which may be through its reducing pathogenic inflammation and antioxidant properties *in vivo* (7, 10–13). Like metformin, rapamycin (sirolimus) is also an FDA-approved mTOR inhibitor and has been found first as an immunosuppressant following renal transplantation (14). Subsequently, rapamycin has been found to extend lifespan and health span in many studies from yeast to monkeys (15–17). Similarly, rapamycin also has beneficial effects on many neurological diseases, such as AD and ischemic stroke in addition to its anti-aging property and anticancer effects through a combination of AMP-activated protein kinase (AMPK)-dependent and -independent mechanisms (13). For example, rapamycin-induced autophagy and prevented cognitive impairment in amyloid-beta (Aβ1-42)-induced AD rat (14). Nicotinamide mono nucleotide (NMN) is one of the main precursors of nicotinamide adenine dinucleotide (NAD^+^) — a vital enzyme for various critical cell functions. Recent studies have shown that boosting NMN levels can increase insulin sensitivity, alleviate mitochondrial dysfunction, extend lifespan, and even reverse age-related conditions by stimulating the NAD^+^ metabolism. Similar to metformin and rapamycin, NMN has been shown with positive effects on the age-associated physiological decline, such as the decrease of insulin sensitivity, eye function, and bone density, without obvious deleterious effects (18). NMN administration protected neurovascular coupling responses in aged mice (19). Subcutaneous administration of NMN could also decrease β-amyloid production, amyloid plaque burden, synaptic loss, and inflammatory responses, as well as regulate the expression of APP cleavage secretase in AD mice (20). Taken together, rapamycin, metformin and NMN are promising anti-aging candidates with effectiveness in treating cognitive dysfunction. VCI predominantly occurs in the elderly and encompasses the full range of cognitive deficits. Therefore, we asked whether these anti-aging drugs could attenuate cognitive impairment and white matter lesions (WMLs) in the rat model of VCI.

In this study, we used bilateral common carotid artery occlusion (BCCAO), which is widely accepted as an experimental model of VCI (21, 22), to examine the effects of the anti-aging drugs on the outcomes after surgery. We found that pretreatment of metformin, rapamycin, or NMN for 14 days could significantly improve the cognitive impairment and reduce the WMLs in BCCAO rats compared with the vehicle group. We also found that pretreatment of metformin, rapamycin, or NMN could reduce microglial activation and the M1 microglia but increase the M2 microglia compared to the vehicle group. Importantly, the BCCAO-induced microglial phagocytosis of myelin was suppressed after the administration of metformin, rapamycin, or NMN. Our data suggest that metformin, rapamycin, or NMN could protect or attenuate cognitive impairment and WMLs by modifying microglial polarization and inhibiting phagocytosis.

## Materials and Methods

### Animals

Adult male Sprague-Dawley rats weighing 250–300 g were obtained from the Experimental Animal Center of Wenzhou Medical University and housed in a specific pathogen-free environment under a 12-h light/12-h dark cycle at 21°C and 50% humidity. Food and water were available *ad libitum*. All animal experiments were approved by the Institutional Animal Care and Use Committee (IACUC) of the Wenzhou Medical University.

### Animal Surgery

Bilateral common carotid artery occlusion (BCCAO; 2-vessel occlusion, 2-VO) in rats has been extensively studied, since they were first found to develop WMLs similar to that in humans (see review (21, 23, 24). Animals were randomly assigned to sham or BCCAO surgery procedures, which was carried out according to a previous study (22). Briefly, the rats were anesthetized with 30 mg/kg of pentobarbital sodium. A neck ventral midline incision of about 1 cm was made, and bilateral common carotid arteries were carefully exposed by separating them from the vagus nerve and their sheath. Both common carotid arteries were occluded with double 4-0 silk sutures, and then the artery was cut between the two ligated silk sutures. The sham group received the same surgical procedure without ligation and cutoff carotid arteries. Rectal temperature was maintained at 36.5–37.2°C during the surgery. The animals were returned to their normal cage to recover with free access to food and water.

### Drug Administration

**Figure 1:**
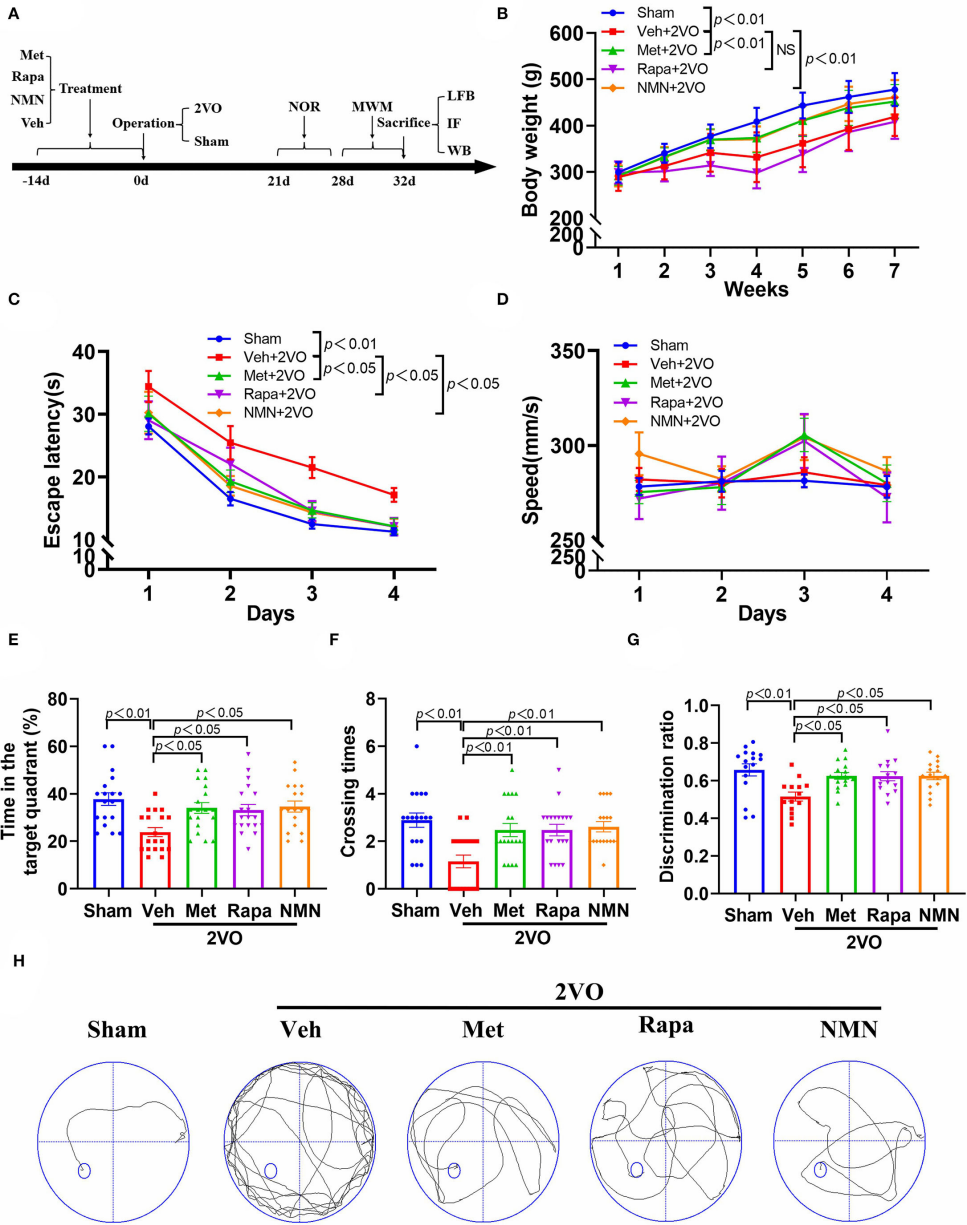
Metformin, rapamycin, or NMN pretreatment improves cognitive impairment in BCCAO rats. **(A)** Experimental protocol. Rats received either saline (Veh), metformin (Met), rapamycin (Rapa), or NMN (NMN) at 2 weeks before surgery *via* intraperitoneal (i.p.) injection daily for 14 consecutive days. The animals were scarified 32 days after surgery. **(B)** The body weight of rats from different groups (*n* = 20). The *p*-values were assessed by two-way repeated-measures ANOVA with Tukey's *post hoc* test. The escape latency **(C)** and swimming speed **(D)** during the training phase, and duration in the target quadrant **(E)**, and the times crossing the area the platform placed in the training phase **(F)** during probe trial of MWM test (*n* = 18–19 per group). The *p*-values in C and D were assessed by two-way repeated-measures ANOVA with Tukey's *post hoc* test. The *p*-values in E and F were assessed by Kruskal–Wallis test with a Dunn post-test. **(G)** The discrimination ratio in novel object recognition test (*n* = 14–16 per group). The *p*-values were assessed by one-way ANOVA with a Tukey's test. **(H)** Representative trajectories in swim path in sham-operated rats and BCCAO rats treated with Veh, Met, Rap, or NMN. All data were shown as mean ± SEM. 2VO, two-vessel occlusion; NOR, novel objective recognition; MWM, Morris water maze; LFB, Luxol fast blue staining; IF, immunofluorescence; WB, Western blot; and NS, not significant.

Before BCCAO, rats were randomly allocated to receive either vehicle (saline), metformin (100 mg/kg; MedChem Express, New Jersey, United States, Cat# HY-B0627), rapamycin (0.25 mg/kg; MedChem Express, New Jersey, United States, Cat# HY-10219), or NMN (100 mg/kg; MedChem Express, New Jersey, United States, Cat# HY-F0004) *via* intraperitoneal (i.p.) injection daily for 14 consecutive days (Figure 1A). BCCAO was performed after injection. Body weights were measured weekly during the whole process of the animal experiment.

### Neurological Behavioral Tests

Neurologic function tests in rats were conducted in a blinded manner 21–32 days after surgery, which included the Novel Object Recognition (NOR) test to assess the recognition memory and the Morris water maze (MWM) test to assess spatial working memory (25).

#### Novel Object Recognition

The NOR test was performed 3 weeks after surgery as described previously (25). Briefly, the rats were placed in a 40 × 40 × 45 cm box to explore the area for 10 min per day for the first 3 days. After an interval of 24 h, two same objects were placed in the two corners of the box. The rat was then placed at the mid-point of the wall opposite both objects with its head pointing away from them. Each rat was allowed to explore the area for 10 min. An hour later, one of the objects was replaced with a new one, and each rat explored the objects for 5 min. Object exploration was defined when the distance between the rat's nose and the object was <2 cm while climbing over or sitting on the object was not included. After each trial, the objects and box were wiped with 75% ethanol to eliminate odor cues for the next rat. The discrimination ratio was calculated as novel object exploration time/ (novel object exploration time + familiar object exploration time).

#### Morris Water Maze

The MWM test was performed in a circular tank with a diameter of 170 cm, containing water at approximately 20–22°C, using the Morris water maze system (Shanghai Jiliang Software Technology Company, Shanghai, China). The swimming pool was virtually divided into four equal quadrants. The edible pigment was used to make the water opaque to camouflage the submerged platform. The hidden circular platform (10 cm in diameter) was submerged 2 cm below the water surface. The MWM test was performed as previously described (26). The rats were given four trials with different designated start positions in each trial per day to explore the platform over consecutive 4 days. If the rat failed to find the platform within 120 s, it would be guided to the platform by a stick and it would stay on the platform for 15s; otherwise, the trial would be finished after the rat remained on the platform for 15 s. The rat which swam around the pool or got to the platform as soon as it was put into the water was excluded. The swimming speed was recorded to exclude rats which had a defect in vision or motor ability. The escape latency was the time the rat spent reaching the platform from the start position. Then on the fifth day, the platform was removed, and the rat was allowed to swim freely for 30 s. The duration was defined as the time the rat spent in the target quadrant where the platform formerly existed and crossing times were the number of times that the rat crossed the target quadrant. All trials were performed in a quiet and dark room.

### Histology and Immunohistochemistry

Rats were euthanized with sodium pentobarbital followed by transcardially perfused with ice-cold saline and 4% paraformaldehyde (PFA) consecutively. The brains were dissected and fixed in 4% PFA overnight. The brains were then dehydrated and immersed in the paraffin wax for paraffin sections or immersed in the OCT for frozen sections.

Luxol fast blue (LFB) staining was performed to stain myelin using Luxol Fast Blue/Cresyl Violet Stain Kit (Cat# G3245, Solarbio, Beijing, China) according to the manufacturer's protocol. The severity of white matter lesion was graded as normal (grade 0), disarrangement of nerve fibers (grade 1), formation of marked vacuoles (grade 2), and disappearance of myelinated fibers (grade 3) (27).

Immunohistochemistry and double immunofluorescent staining were performed on the frozen or paraffin sections by incubation with primary antibodies at 4°C overnight as previously described (28). The primary antibodies used were as follows: rabbit anti-myelin basic protein (MBP) (Cat# ab40390, Abcam, United States; 1:500), mouse anti-SMI32 (Cat# 801701, Biolegend, United States; 1:1000), goat anti-ionized calcium-binding adaptor molecule 1 (Iba1) (Cat# 011-27991, Wako, United States; 1:300), rabbit anti-CD68 (Cat# ab283654, Abcam, United States; 1:100), mouse anti-CD206 (Cat# sc-58986, Santa Cruz Biotechnologies, United States; 1:50), and mouse anti-MBP (Cat# BF8010, Affinity, United States; 1:100). After washing with PBS, the sections were incubated with the corresponding secondary antibodies at 37°C for 1 h. The secondary antibodies used were as follows: Alexa Fluor 350-, 488-, 594-, or 647-conjugated donkey anti-rabbit, anti-mouse, or anti-goat IgG (1:1000; Abcam, United States).

The fluorescence intensity of the SMI32/MBP ratio was recorded by a microscope (Leica, Germany) and quantified by ImageJ (NIH, United States). The quantification of immunostaining positive cells in the striatum was also quantified by a researcher blinded to the experiment design. The data were presented as the number of positive cells and percent-stained area per field, respectively (28).

### Western Blot

Western blot was performed according to the previous study (29). The proteins were extracted from the striatum and the concentrations were determined by the BCA Protein Assay kit (Thermo Scientific, United States). The protein (30 μg) was loaded onto 12% SDS-PAGE (Solarbio, Beijing, China) and subsequently transferred onto a polyvinylidene difluoride (PVDF) membrane (Merck Millipore, United States). The blot was incubated in blocking buffer (5% milk in Tris-buffered saline with 0.1% Tween-20) and then incubated with rabbit anti-MBP (Cat# ab40390, Abcam, United States; 1:1,000) or rabbit anti–β-actin (1:1,000, Catalog No. 4970S; Cell Signaling Tech) overnight at 4 °C. After washing with TBST (0.01% Tween 20 in TBS), the blots were incubated with horseradish peroxidase-conjugated IgG secondary antibody (Biosharp, BL003A, 1:10,000) for 1 h at room temperature and then reacted with BeyoECL Star (Beyotime, Shanghai, China). The chemiluminescence results were recorded by Amersham Imager 680 imaging system (CTL, United States) and analyzed by ImageJ (NIH, United States).

### Statistical Analysis

All values were expressed as mean ± standard error of mean (SEM). The data normality was determined using a Shapiro–Wilk's test. For normally distributed populations of data points, a one-way analysis of variance (ANOVA) followed by Tukey's *post-hoc* test were used. For data that failed the normality test, a Kruskal–Wallis test with a Dunn post-test were used. The results of escape latency and swimming speed from the MWM test were compared by two-way repeated measurements analysis of variance (ANOVA) followed by Tukey's *post-hoc* test. All statistical analyses were performed by GraphPad Prism version 8.02 (Graphpad Prism Software, San Diego, CA), and *p*<0.05 was considered statistically significant.

## Results

### Metformin, Rapamycin, or NMN Pretreatment Attenuates Cognitive Impairment After BCCAO

First, we investigated whether pretreatment of the anti-aging drugs could improve cognitive impairment in rats after BCCAO (2VO). As indicated in Figure 1A, rats were pretreated with either vehicle, metformin, rapamycin, or NMN *via* intraperitoneal (i.p.) injection daily for 14 consecutive days before surgery. All rats exhibited gradual increases in body weight over time (Figure 1B). However, after surgery, rats pretreated by vehicle or rapamycin had significantly lower body weights compared with the sham group. Rats pretreated with metformin, or NMN had higher body weights than rats pretreated with vehicle. We used the MWM task to test spatial learning and memory 28–32 days after BCCAO. We found that the BCCAO rats exhibited learning and memory impairment compared to the sham-operated rats, as manifested by significantly longer escape latencies (loss of spatial learning) (Figure 1C) and decreased time in the target quadrant (loss of spatial memory) (Figure 1F). However, spatial learning and memory dysfunction were reversed in the BCCAO rats pretreated with metformin, rapamycin, or NMN, compared with the vehicle group (Figures 1C,E,F,H). To further confirm the findings, new object recognition test was also performed. As shown in Figure 1G, pretreatment of metformin, rapamycin, or NMN also alleviated cognitive impairment after BCCAO by the analysis of the new object recognition test. However, no significant difference was found between groups pretreated with metformin, rapamycin, or NMN. Rats from all groups exhibited similar swim speeds, suggesting that the memory tests were not confounded by major differences in swimming abilities (Figure 1D).

Taken together, our findings indicate that these three drugs effectively prevent BCCAO-induced cognitive impairment in rats.

### Metformin, Rapamycin, or NMN Pretreatment Attenuates White Matter Lesions After BCCAO

**Figure 2:**
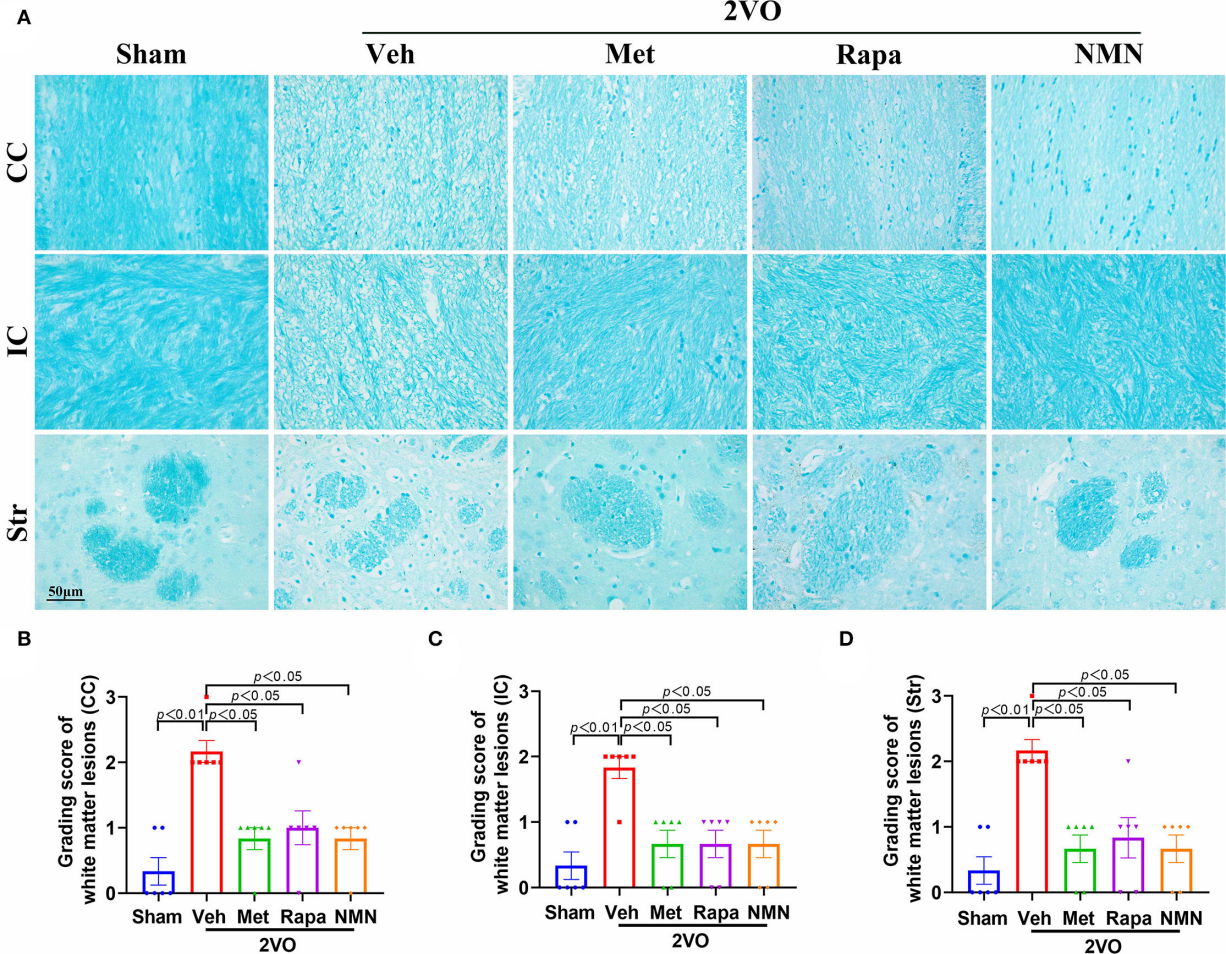
Metformin, rapamycin, or NMN pretreatment ameliorates WMLs in BCCAO rats. **(A)** Representative images of Luxol fast blue (LFB) staining in the corpus callosum (CC), internal capsule (IC), and striatum (Str) in BCCAO or sham rats pretreated with vehicle (Veh), metformin (Met), rapamycin (Rapa), or NMN (NMN) at 2 weeks before surgery. **(B–D)** Quantitative analysis of WMLs in BCCAO or sham-operated rats after treatment. The extent of WMLs was graded as normal (Grade 0), disarrangement of nerve fibers (Grade 1), formation of marked vacuoles (Grade 2), and loss of myelinated fibers (Grade 3). All data were shown as mean ± SEM, *n* = 6 per group. The *p*-values were assessed by Kruskal–Wallis test with a Dunn post-test. 2VO, two-vessel occlusion.

Next, we tried to explore the mechanisms underlying protecting cognitive deficits in the BCCAO rats pretreated with the anti-aging drugs. Accumulating evidence demonstrates that white matter integrity is essential for maintaining cognitive function. White matter is mainly composed of axons, myelin sheath around the axon, and oligodendrocytes participating myelination. Therefore, we assessed the white matter integrity with Luxol fast blue staining, which is designed for staining myelin/myelinated axons. A shown in Figure 2, the white matter was significantly damaged in the corpus callosum, internal capsule, and striatum 32 days after BCCAO, as evidenced by reduced LFB staining, along with severe rarefaction and marked vacuole. Pretreatment of metformin, rapamycin, or NMN for 14 days significantly ameliorated BCCAO-induced myelin disarrangement and damage, compared with the vehicle group (Figure 2).

**Figure 3:**
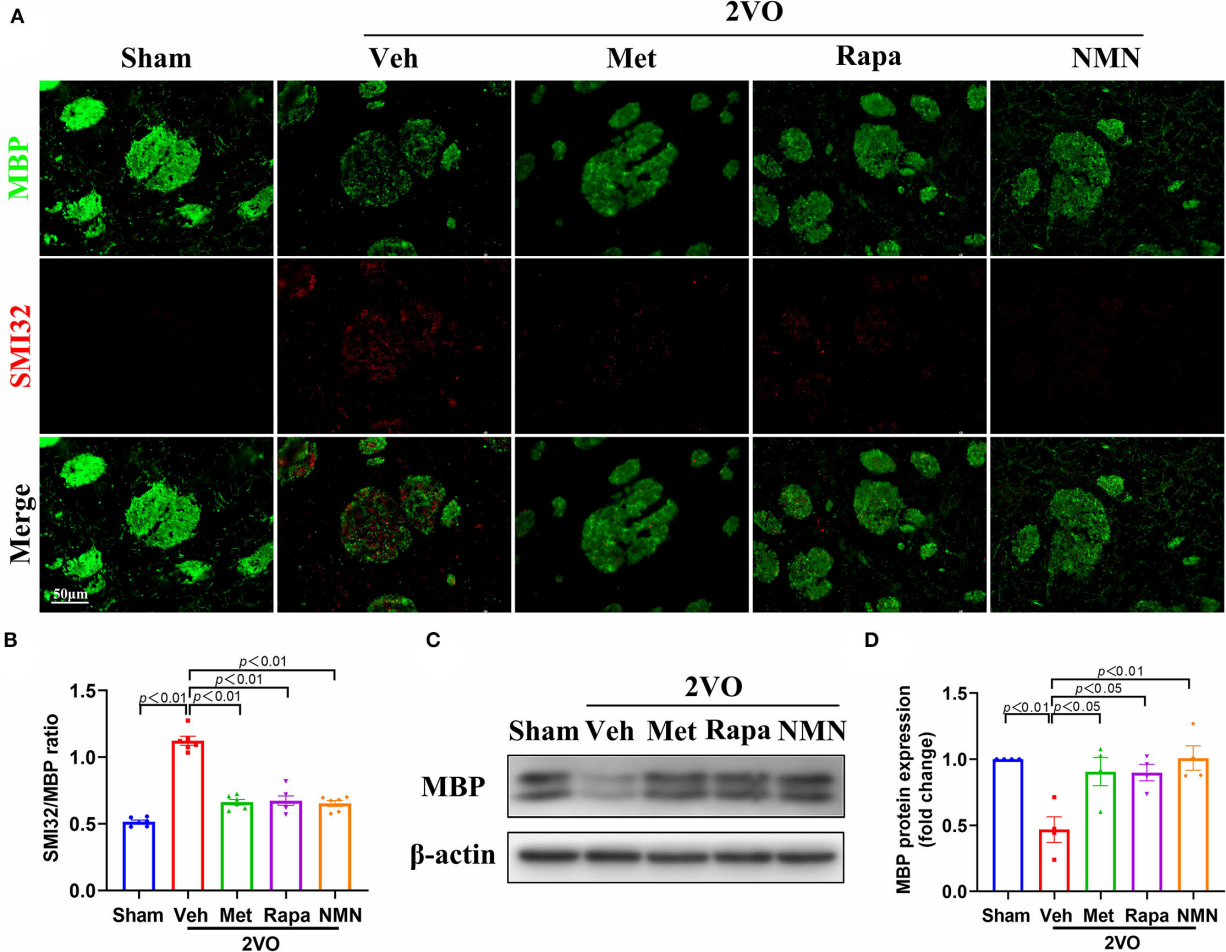
Metformin, rapamycin, or NMN pretreatment improves axonal and oligodendrocyte damage induced after BCCAO. **(A)** Representative images of dephosphorylated neurofilament protein (SMI32, red) and MBP (green) in striatum 32 days after BCCAO in rats. **(B)** Quantification of the ratio of SMI32 to MBP fluorescence intensity in the striatum, illustrated as fold-change compared with the sham average value. *n* = 6 per group. The *p*-values were assessed by one-way ANOVA with a Tukey's test. **(C)** Western blot analysis of MBP protein from the striatum in sham and BCCAO rats after treatment. **(D)** Quantitative analysis of MBP protein from the striatum in each group. MBP protein was normalized to the β-actin level of the same sample (*n* = 4 per group). The *p*-values were assessed by one-way ANOVA with a Tukey's test. All data were shown as mean ± SEM. Met, metformin; Rapa, rapamycin; NMN, nicotinamide mononucleotide; Veh, vehicle; 2VO, two-vessel occlusion.

WMLs were further evaluated by immunofluorescence using MBP (a marker for myelin) and SMI32 (a marker for demyelinated axons). As expected, BCCAO induced the disruption in myelin sheaths and axon damage, as identified by a decrease of MBP levels and a concurrent increase in SMI32 intensity and SMI32/MBP ratio in the striatum 32 days after BCCAO, compared to the sham-operated rats (Figure 3). After pretreatment with metformin, rapamycin, or NMN, the SMI32 intensity and SMI32/MBP ratio were significantly reduced in the BCCAO rats compared with the vehicle group (Figure 3) and prevented the loss of MBP. Western blot confirmed that the expression level of MBP protein was reduced in BCCAO rats, which, however, was improved after metformin, rapamycin, or NMN pretreatment (Figures 3C,D). Collectively, these results demonstrate that these anti-aging drugs ameliorated BCCAO-induced demyelination and axonal damage in white matter tracts, which may correlate with cognitive impairment in the rat model of VCI.

### Metformin, Rapamycin, or NMN Pretreatment Influences BCCAO-Induced Microglial Response

**Figure 4:**
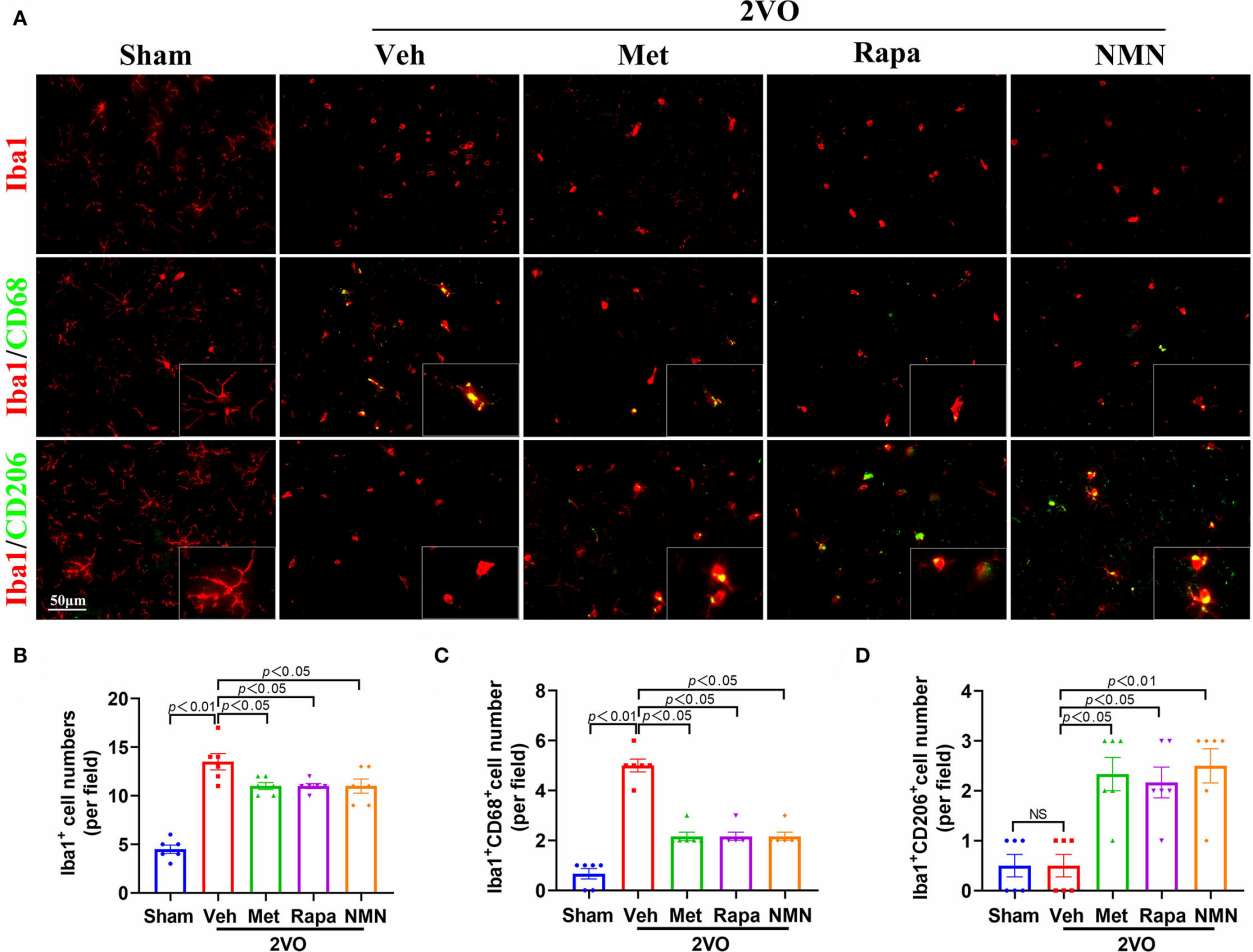
Metformin, rapamycin, or NMN pretreatment suppresses BCCAO-induced microglial response. **(A)** Representative images of immunostaining of Iba1^+^ microglia (red, top panel), CD68^+^ (green, middle panel) and CD206^+^ (green, bottom panel) in striatum of sham or BCCAO rats pretreated with vehicle (Veh), metformin (Met), rapamycin (Rapa), or NMN (NMN) at 2 weeks before surgery. Quantitative analysis of Iba1^+^ cells **(B)**, Iba1^+^CD68^+^ cells **(C)** and Iba1^+^CD206^+^ cells **(D)** in striatum in sham or BCCAO rats after pretreatment. The *p*-values in A were assessed by one-way ANOVA with a Tukey's test. The *p*-values in B and C were assessed by Kruskal–Wallis test with a Dunn post-test. All data were shown as mean ± SEM, *n* = 6 per group. 2VO, two-vessel occlusion; NS, not significant.

Microglia are the main resident macrophages in CNS and play a significant role in WMLs. Indeed, we found that the number of Iba1^+^ microglia was substantially increased in the striatum 32 days after BCCAO, compared to the sham-operated rats (Figure 4). These microglia were immunopositive for CD68, which is a lysosomal protein expressed at high levels by activated microglia with M1 phenotype, suggesting increased activated classic proinflammatory type (M1) microglia after BCCAO, which primarily accumulated in the striatum. The M2-associated proteins (CD206) were reduced in the BCCAO rats compared to the sham-operated rats (Figure 4). After pretreatment with metformin, rapamycin, or NMN, the number of Iba1^+^ microglia and Iba1^+^/CD68^+^ microglia was significantly reduced in the striatum, compared with the vehicle group. However, the number of Iba1^+^/CD206^+^ M2 microglia was increased after the anti-aging drug pretreatment in the BCCAO rats, compared with the vehicle-treated group (Figure 4). These data suggest that metformin, rapamycin, or NMN significantly impact the microglial activation and polarization in BCCAO rats.

### Metformin, Rapamycin, or NMN Pre-treatment Suppresses BCCAO-Induced Microglial Phagocytosis

**Figure 5:**
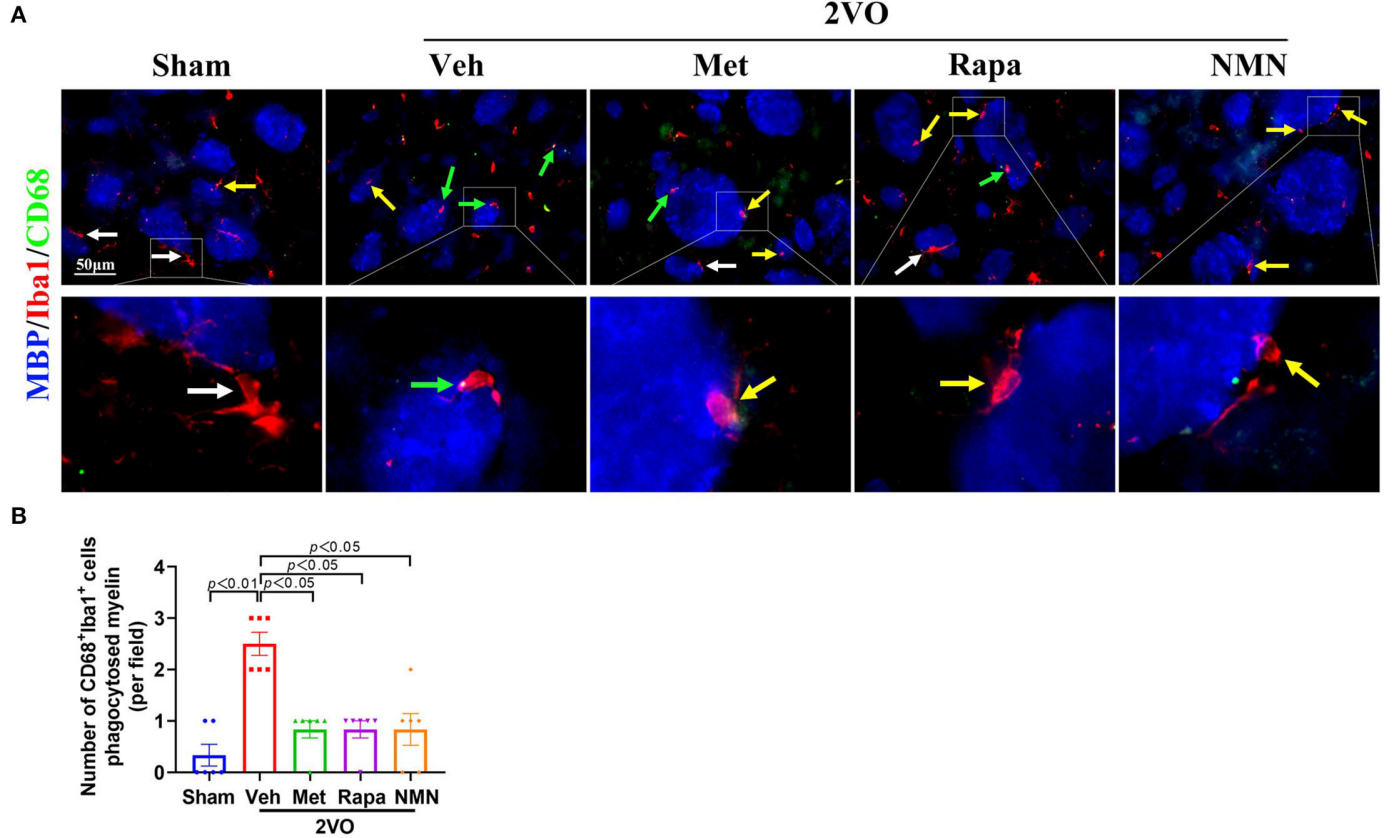
Metformin, rapamycin, or NMN pretreatment alleviates microglial phagocytosis of myelin in the striatum after BCCAO. **(A)** Photomicrographs of triple immunofluorescent staining for Iba1 (red), CD68 (green), and MBP (blue). White arrows indicate Iba1^+^ microglia. Yellow arrows indicate Iba1^+^CD68^+^ microglia. Green arrows indicate Iba1^+^CD68^+^ MBP^+^ microglia. **(B)** Quantification of CD68 (green), Iba1 (red), and MBP (blue)-positive cells in the striatum of sham-operated and BCCAO rats pretreated with vehicle (Veh), metformin (Met), rapamycin (Rapa), or NMN (NMN) for 2 weeks before surgery. The *p*-values were assessed by the Kruskal–Wallis test with a Dunn post-test. All data were shown as mean ± SEM. *N* = 6 per group. 2VO, two-vessel occlusion.

Finally, we investigated the link between microglial activation and phagocytosis. Proinflammatory M1 microglia-mediated phagocytosis could phagocytose live neurons, neurites, or myelin, which thus is detrimental. We performed triple-label immunofluorescence staining for Iba1, CD68, and MBP. The triple-immunopositive (Iba1^+^/CD68^+^/MBP^+^) microglia were observed in the striatum 32 days after BCCAO. Confocal images show that MBP was localized with Iba1^+^CD68^+^ microglia (Figure 5). In contrast, the quantification analysis showed that the number of colocalized Iba1^+^/CD68^+^/MBP^+^ microglia was reduced in BCCAO rats pretreated with metformin, rapamycin, or NMN, compared with the vehicle group (Figure 5). These data indicate that BCCAO significantly induced the microglial phagocytosis of myelin in the white matter regions, which, however, could be suppressed with pretreatment of metformin, rapamycin, or NMN.

## Discussion

This study proved that pretreatment of the anti-aging drugs metformin, rapamycin, or NMN could prevent cognitive impairment and WMLs in BCCAO rats through reducing microglial activation and polarization and preventing microglial phagocytosis of myelin. These results reveal the therapeutic potential of anti-aging drugs in the recovery of cognitive function and protection and/or repair of WMLs in VCI.

Although there is no definitive pathological definition of VCI, it is generally accepted that a cerebrovascular disease (CVD) results in global and/or local cerebral hypoperfusion leading to cortical and subcortical infarcts and white matter lesions (WMLs) (30, 31). Chronic cerebral hypoperfusion (CCH)-induced WMLs are thus considered to be a key mechanism leading to VCI and dementia (32–34). Emerging evidence confirms that WMLs underlie much of the memory impairment in the cognitive decline of VCI. Consistently, we found that the MBP level in the striatum decreased in BCCAO rats, compared with the controls, demonstrating that BCCAO induced myelin loss and sustained to at least 4 weeks, along with cognitive impairment based on the MWM test and novel object recognition test. We also found that the cognitive impairment was significantly improved in BCCAO rats pretreated with anti-aging drugs compared with the vehicle group, in parallel with ameliorating BCCAO-induced WMLs, as evidenced by immunostaining using myelin marker and demyelinated axon, which were further confirmed by histology. Therefore, metformin, rapamycin, or NMN confer neuroprotection against WMLs and cognitive impairment induced by BCCAO in rats.

Although, there is a lack of mechanistic insight into the evolution and progress of VCI, several studies have shown that inflammatory response may play a critical role in WMLs after CCH (35). First, inflammatory cells, such as microglia, are activated and accumulated within the hippocampal region and white matter in parallel with the evolving damage to myelinated axons (27, 36–39). For example, microglia were activated in the white matter in aged rhesus monkeys, and most of them showed phagocytic phenotypes (40). Second, this inflammatory reaction has been suggested to lead to the development of WMLs through the release of proinflammatory factors (41–43). In fact, the inhibition of microglial or glial activation attenuated WMLs and restored white matter function in animal models of CCH (44–47). Yet, microglia emerge as central players in brain disease (48). Third, disruption of axon–glial integrity and proliferation of microglia are indicated to be strongly associated with impairment in working memory (49, 50). These data suggest that neuroinflammatory processes, which are mainly mediated by activated microglia, are crucial for the initiation and progression of VCI (35, 51). Consistently, we found that metformin, rapamycin, or NMN pretreatment could modify the informatory responses, including a reduction in the microglial activation and an increase in the microglial polarization from M1 to M2 phenotypes. Indeed, recent studies show that the microglia are increased or activated in rodent models of BCCAO (36, 52, 53) and post-mortem human brain of VCI (35). Microglia could clear the myelin debris in old rhesus monkeys (40). While the accumulation of myelin debris may also inhibit oligodendrogenesis and myelination. Similarly, pro-inflammatory M1 microglia could also attenuate the clearance of myelin debris (54). Consistently, previous studies have shown that Iba1^+^CD68^+^ microglia are strongly associated with WMLs, because these cells could phagocyte myelin fibers rather than myelin debris (55). CD206^+^ M2 microglia has been found with a positive effect on debris clearance, damage repairment, and axonal regeneration (56, 57). Administration of microglial inhibitor restores white matter function related to cognitive function (44–46). Therefore, VCI is strongly associated with inflammation, reduction of remyelination, and microglial activation. Metformin, rapamycin, or NMN could protect the central nervous system (CNS) diseases *via* regulating the activities of microglia (58–60). These data suggest that anti-inflammatory effects are one of the mechanisms underlying anti-aging drug-mediated protection of cognitive dysfunction and WMLs after BCCAO in rats.

**Figure 6:**
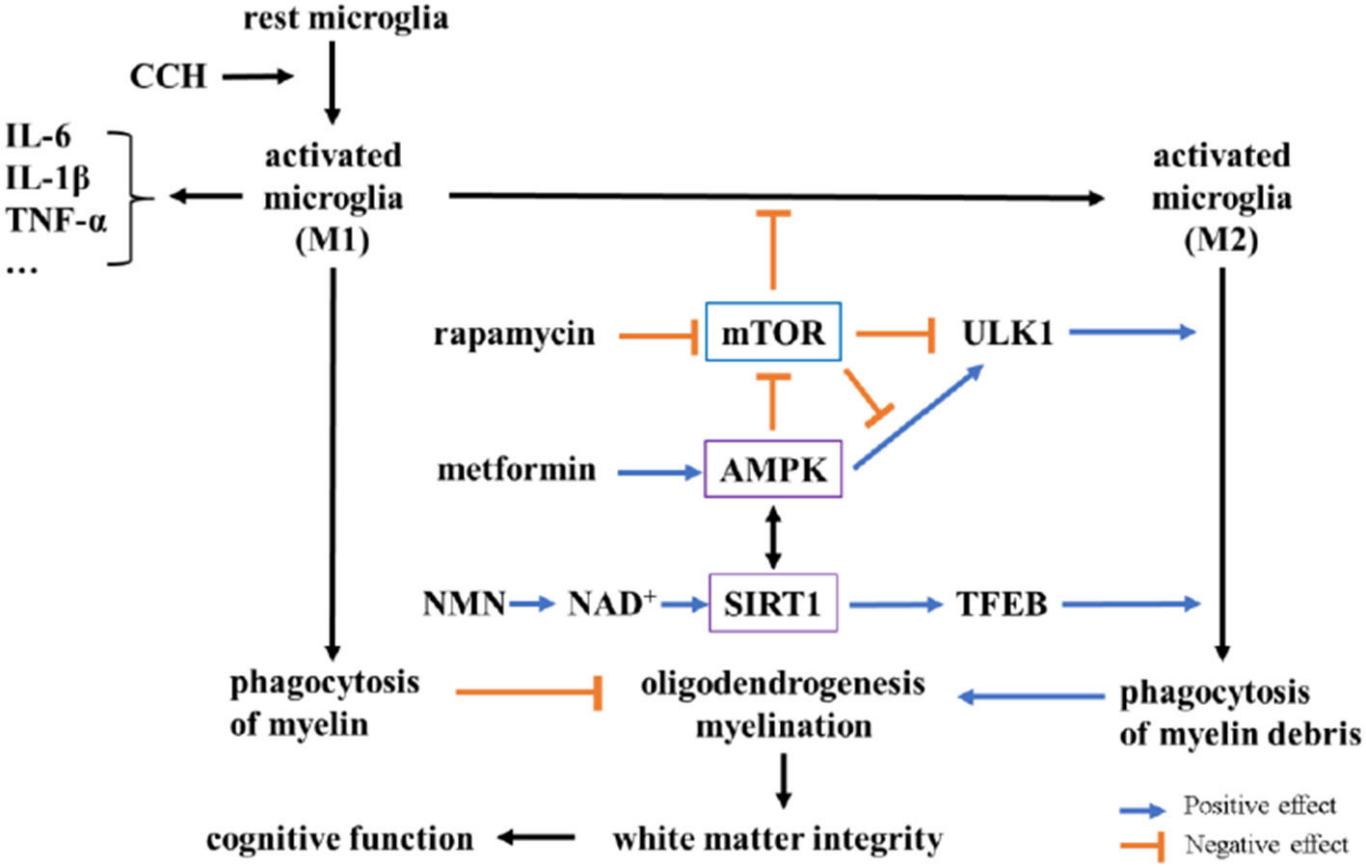
Potential mechanisms underlying neuroprotection of metformin, rapamycin, and NMN after BCCAO in rats. In chronic cerebral hypoperfusion (CCH) rats, activated microglia (M1) increase and destroy white matter integrity by releasing pro-inflammatory factors (like IL-6, IL-1β) and inhibiting oligodendrogenesis and myelination *via* microglial phagocytosis. Metformin and NMN could activate AMP-activated protein kinase (AMPK) and Sirtuin 1 (SIRT1), respectively. Rapamycin inhibits mTOR to increase M2 microglial polarization. AMPK and SIRT1 could also inhibit M1 polarization by decreasing the expression of NF-κB, as well as promoting M2 polarization. The activation of AMPK and the inhibition of mTOR could promote phagocytosis *via* the phosphorylation of ULK1, while SIRT1 could promote phagocytosis through deacetylation of transcription factor EB (TFEB). There is a complex interplay among AMPK, mTOR, and SIRT1.

Previous studies reported the neuroprotective effects of metformin in CNS diseases including stroke, AD, and PD (61–63). In neurodegenerative diseases, metformin could make a positive effect by activating AMPK signaling, downregulating mTOR pathway, modulating glucose metabolism, and enhancing mitochondriogenesis and autophagy (64). Everolimus, an analog of rapamycin, could regulate the balance of microglial M1/M2 phenotypes by inhibiting the activity of mTORC1 in bilateral common carotid artery stenosis (BCAS) mice (59). Similarly, rapamycin could also promote M1 to M2 switch of microglia *via* upregulating autophagy (65). Although there is little evidence about the interaction of NMN with microglia, NAD^+^ and NAD^+^ precursor, nicotinamide riboside (NR), have similar effects. NAD^+^ treatment in mice with multiple sclerosis could reduce demyelination, aberrant activation of microglia, and motor dysfunction through activating autophagy (66). In AD mice, NR administration increased NAD^+^ level in the brain and decreased the expression level of pro-inflammatory cytokines, as well as reduced the activation of microglia and astrocytes (60). Consistent with these findings, our study found that metformin, rapamycin, or NMN pretreatment increased the Iba1^+^CD206^+^ microglia (M2) but reduced the Iba1^+^CD68^+^ microglia (M1), suggesting that these anti-aging drugs could polarize M1 microglia to the M2 phenotype in the rat model of VCI. Given that the impact of these three drugs on BCCAO had no significant difference, we speculate that the underlying mechanism of these three drugs was similar. For example, both metformin and rapamycin are mTOR inhibitor. It is now known that NMN also activates AMP-activated protein kinase (AMPK). For example, Sirtuin 1 (SIRT1) is an NAD^+−^dependent deacetylase, and NMN supplementation could activate SIRT1 by improving NAD^+^ level (67). Further, SIRT1 has been found to promote autophagy by activating AMPK (68). The interplay between mTOR, and AMPK signaling pathways has been well-documented. Importantly, mTOR/AMPK pathways involve in neuroinflammation including microglial activation. Thus, metformin, rapamycin, and NMN could finally contribute to microglial response through the interaction of AMPK, mTOR, and SIRT1 (Figure 6). Notably, although metformin, rapamycin, or NMN pretreatment could improve cognitive impairment and reduce WMLs after BCCAO, no significant difference is found between the groups treated with metformin, rapamycin, or NMN. The underlying mechanism may be these drugs share similar signaling pathway. It is unclear whether additive effect will be achieved when two or more combined chemicals are applied. We will further determine whether the combined effect of two or more drugs is equal to the sum of the effect of each drug given alone. Previous studies have documented that taking metformin for a long time might lead to vitamin B12 deficiency and anemia in non-diabetic patients (69). Moreover, the safety of taking rapamycin remains controversial up to now (70). Besides, a clinical trial has found that rapamycin had no significant influence on the cognitive function in old people (71). Thus, further studies should be conducted to address these concerns. In comparison, NMN might be safer than metformin and rapamycin. As a clinical trial showed that single oral administration of NMN was safe in healthy Japanese men (72). In addition, chronic administration of NMN in mice also showed few side effects (18). Nevertheless, the expensive price of NMN blocks its widespread use.

In conclusion, our data showed that long-term metformin, rapamycin, or NMN pretreatment significantly ameliorated BCCAO-induced WMLs and profoundly facilitated cognitive functional recovery. Furthermore, AMPK-dependent and -independent microglial activation and phagocytosis played an important role in mediating anti-aging drug neuroprotection.

## Data Availability Statement

The original contributions presented in the study are included in the article/supplementary material, further inquiries can be directed to the corresponding author/s.

## Ethics Statement

The animal study was reviewed and approved by Institutional Animal Care and Use Committee (IACUC) of Wenzhou Medical University.

## Author Contributions

MY and KJ conceived and designed the study and contributed to manuscript writing. MY, XZ, and FC performed the experiments and data analysis. QZ and KJ supervised the study. QZ and BS provided relevant information and revised the manuscript. All authors contributed to the article and approved the submitted version.

## Funding

This work was supported by Zhejiang Provincial Key Discipline-Neurobiology (437201203).

## Conflict of Interest

The authors declare that the research was conducted in the absence of any commercial or financial relationships that could be construed as a potential conflict of interest.

## Publisher's Note

All claims expressed in this article are solely those of the authors and do not necessarily represent those of their affiliated organizations, or those of the publisher, the editors and the reviewers. Any product that may be evaluated in this article, or claim that may be made by its manufacturer, is not guaranteed or endorsed by the publisher.
